# Supplementary material for: Biomarkers for prediction of neurological complications after acute Stanford type A aortic dissection: A systematic review and meta-analysis
Source: PLoS One. 2023 Feb 8;18(2):e0281352. doi: 10.1371/journal.pone.0281352 (PMC9907800; doi:10.1371/journal.pone.0281352)
Supplement: S1 Table — (DOCX) [file pone.0281352.s002.docx]

S1 Table. Detailed risk assessment of publication bias.

| Author | Year | Study population selection | | | |  | Comparability between groups |  | Exposure or Outcome Evaluation | | | NOS Score |
| --- | --- | --- | --- | --- | --- | --- | --- | --- | --- | --- | --- | --- |
|  |  | ① | ② | ③ | ④ |  | ⑤ |  | ⑥ | ⑦ | ⑧ |  |
| Lang Q et al ^8^ | 2021 | 1 | 1 | 0 | 0 |  | 1 |  | 1 | 1 | 1 | 6 |
| Zhang, K et al ^6^ | 2021 | 1 | 1 | 0 | 1 |  | 1 |  | 1 | 1 | 1 | 7 |
| Wan Z et al ^3^ | 2021 | 1 | 1 | 0 | 1 |  | 1 |  | 1 | 1 | 1 | 7 |
| Lv X et al ^9^ | 2021 | 1 | 1 | 0 | 1 |  | 2 |  | 1 | 1 | 1 | 8 |
| Peng X et al ^4^ | 2020 | 1 | 1 | 0 | 1 |  | 0 |  | 1 | 1 | 1 | 6 |
| Kimura F et al ^5^ | 2020 | 1 | 1 | 0 | 1 |  | 1 |  | 1 | 1 | 1 | 7 |
| Fang M et al ^10^ | 2015 | 1 | 1 | 0 | 1 |  | 1 |  | 1 | 1 | 1 | 7 |
| Zhang Z et al ^11^ | 2012 | 1 | 1 | 0 | 1 |  | 1 |  | 1 | 1 | 1 | 7 |
| Dalyanoglu H et al ^12^ | 2012 | 1 | 1 | 0 | 1 |  | 1 |  | 1 | 1 | 1 | 7 |
| Zou Y et al ^13^ | 2011 | 1 | 1 | 0 | 1 |  | 2 |  | 1 | 1 | 1 | 8 |
| Chen B et al ^14^ | 2009 | 1 | 0 | 0 | 1 |  | 2 |  | 1 | 1 | 1 | 7 |
| Liu X et al ^15^ | 2007 | 1 | 0 | 0 | 1 |  | 1 |  | 1 | 1 | 1 | 6 |

Note: ① Whether the case is appropriate ② Representation of cases ③ Selection of controls ④ Determination of controls ⑤ Consider the comparability of cases and controls in design and statistical analysis non-response rate ⑥ Determination of exposure factors ⑦ Determination of exposure factors for cases and controls using the same method ⑧ Non-response rate; NOS, Newcastle-Ottawa Scale
