# Supplementary figures and images for: Biomarkers for prediction of neurological complications after acute Stanford type A aortic dissection: A systematic review and meta-analysis
Source: PLoS One. 2023 Feb 8;18(2):e0281352. doi: 10.1371/journal.pone.0281352 (PMC9907800; doi:10.1371/journal.pone.0281352)

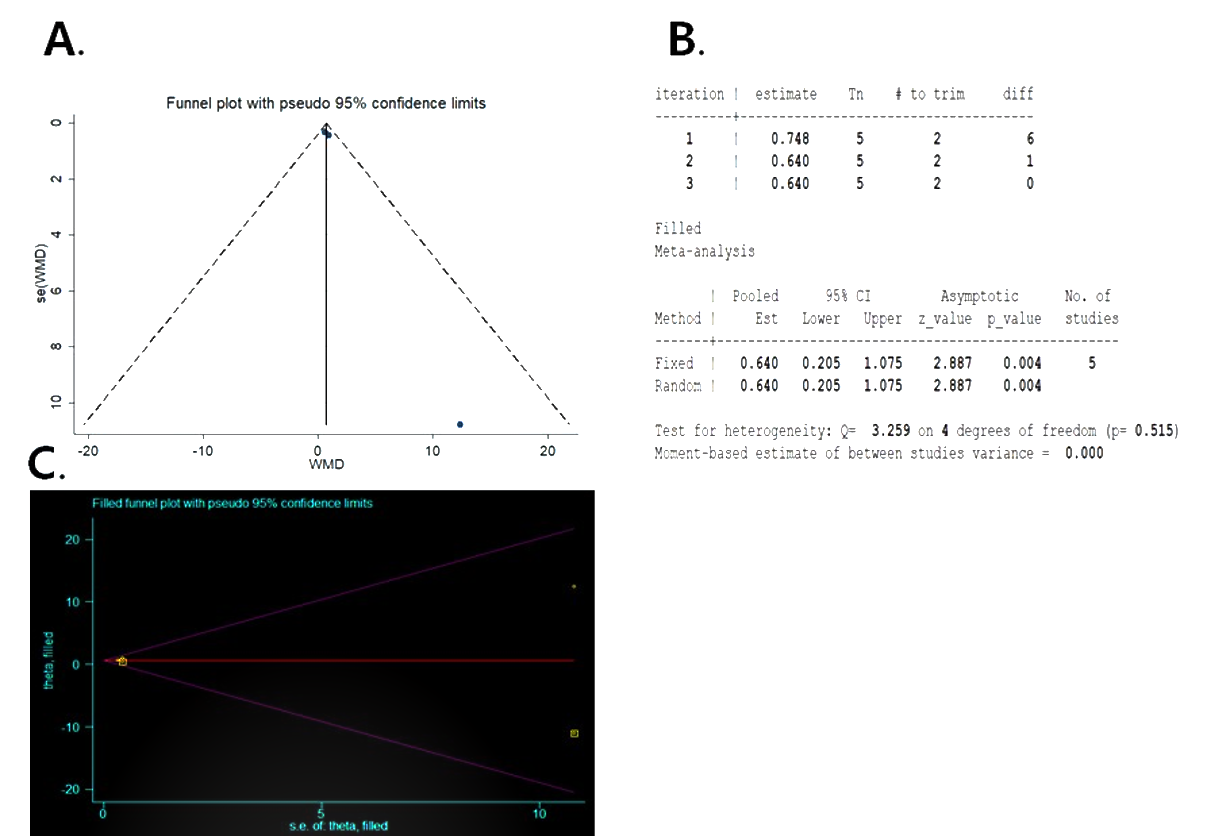

Supplement: S1 Fig — (A) Funnel plot after subgroup analysis. (B) Analysis results after correction by shear compensation method. (C) Funnel plot corrected by trim and filling method. (TIF) [file pone.0281352.s003.tif]

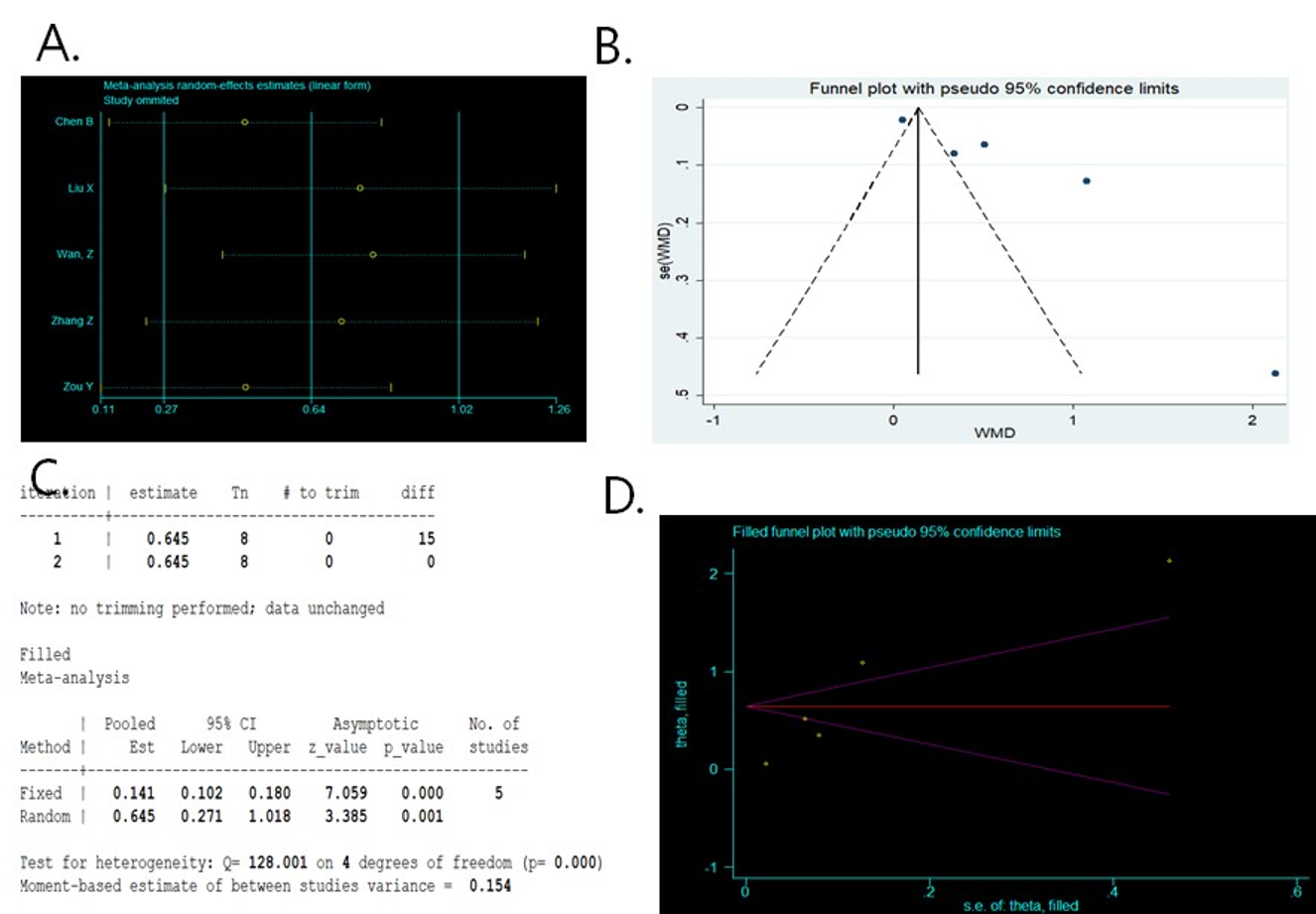

Supplement: S2 Fig — (A) Sensitivity analysis plot. (B) Funnel plot. (C) Analysis results after correction by trim and filling method. (D) Funnel plot corrected by trim and filling method. (TIF) [file pone.0281352.s004.tif]

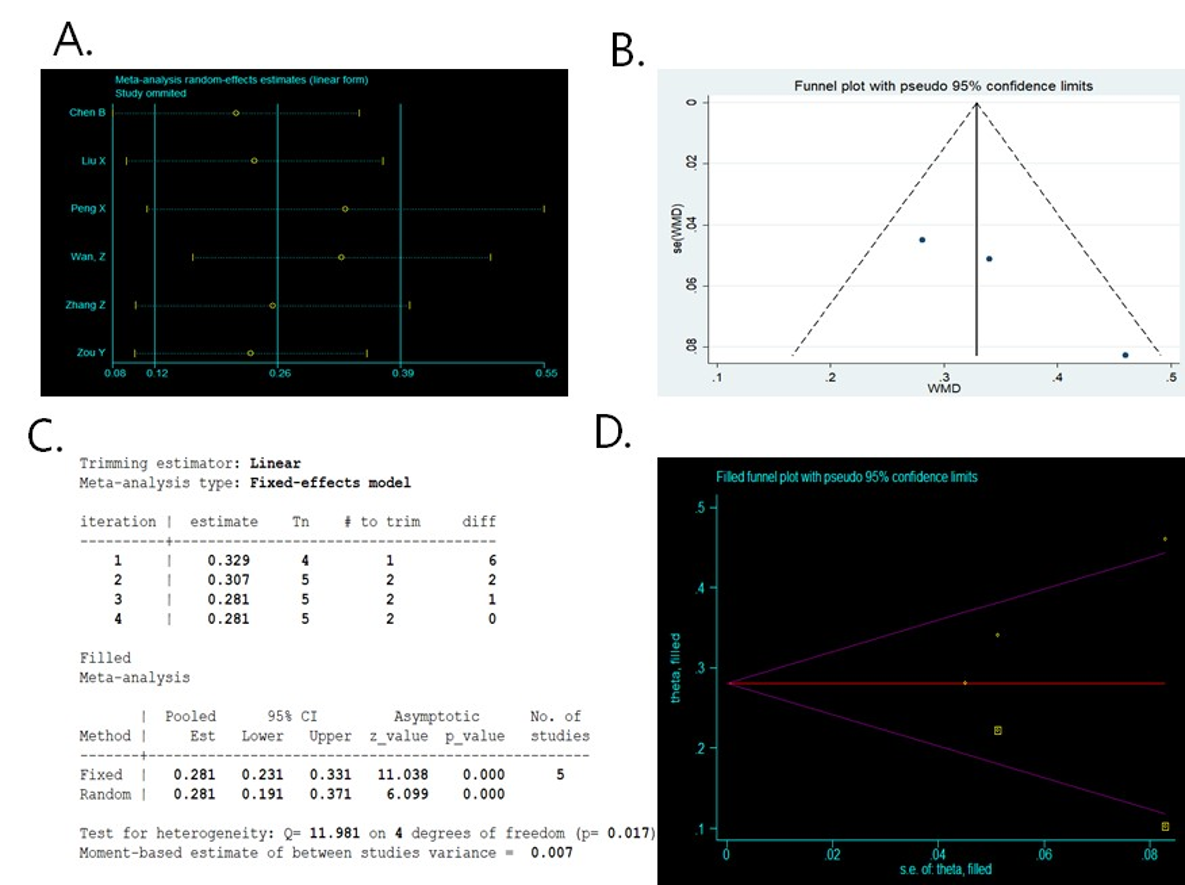

Supplement: S3 Fig — (A) Sensitivity analysis plot before correction. (B) Funnel plot after sensitivity analysis. (C) Analysis results after correction by trim and filling method.(D) Funnel plot corrected by trim and filling method. (TIF) [file pone.0281352.s005.tif]

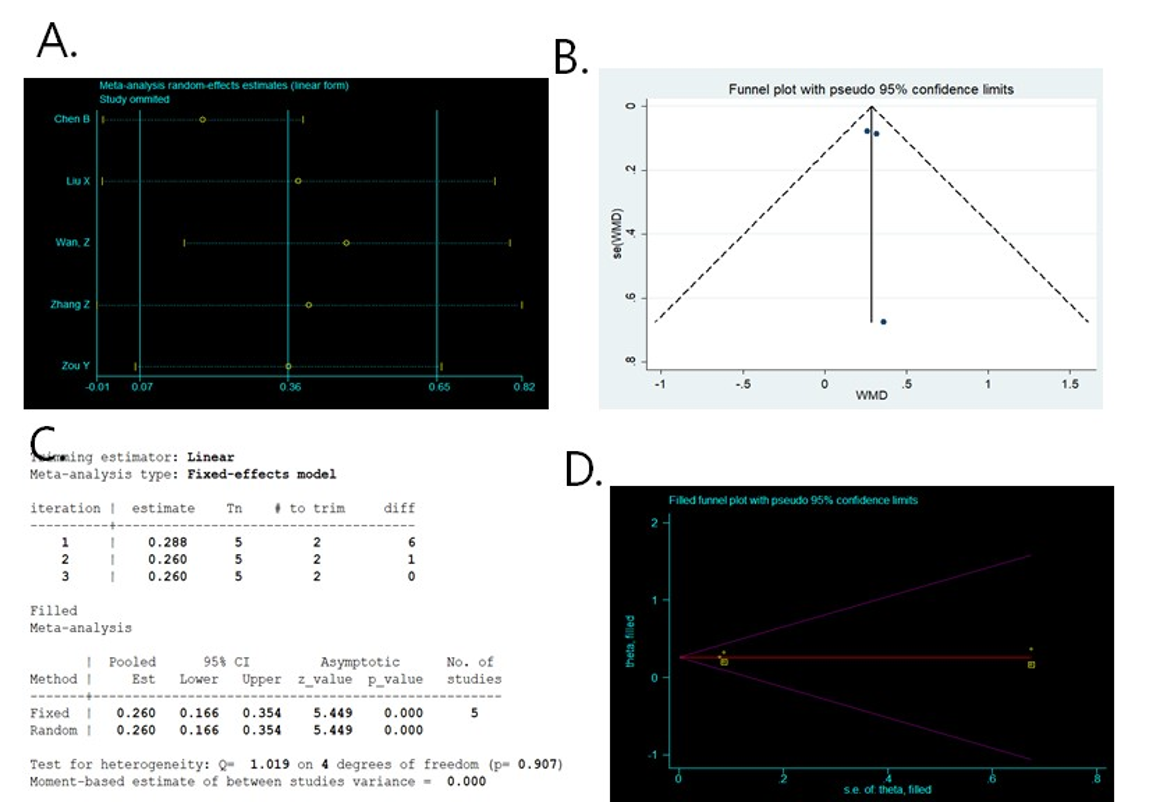

Supplement: S4 Fig — (A) Sensitivity analysis plot before correction. (B) Funnel plot after sensitivity analysis. (C) Analysis results after correction by trim and filling method. (D) Funnel plot corrected by trim and filling method. (TIF) [file pone.0281352.s006.tif]
